# RNF26 Temporally Regulates Virus-Triggered Type I Interferon Induction by Two Distinct Mechanisms

**DOI:** 10.1371/journal.ppat.1004358

**Published:** 2014-09-25

**Authors:** Yue Qin, Mao-Tian Zhou, Ming-Ming Hu, Yun-Hong Hu, Jing Zhang, Lin Guo, Bo Zhong, Hong-Bing Shu

**Affiliations:** State Key Laboratory of Virology, Medical Research Institute, College of Life Sciences, Wuhan University, Wuhan, China; Harvard Medical School, United States of America

## Abstract

Viral infection triggers induction of type I interferons (IFNs), which are critical mediators of innate antiviral immune response. Mediator of IRF3 activation (MITA, also called STING) is an adapter essential for virus-triggered IFN induction pathways. How post-translational modifications regulate the activity of MITA is not fully elucidated. In expression screens, we identified RING finger protein 26 (RNF26), an E3 ubiquitin ligase, could mediate polyubiquitination of MITA. Interestingly, RNF26 promoted K11-linked polyubiquitination of MITA at lysine 150, a residue also targeted by RNF5 for K48-linked polyubiquitination. Further experiments indicated that RNF26 protected MITA from RNF5-mediated K48-linked polyubiquitination and degradation that was required for quick and efficient type I IFN and proinflammatory cytokine induction after viral infection. On the other hand, RNF26 was required to limit excessive type I IFN response but not proinflammatory cytokine induction by promoting autophagic degradation of IRF3. Consistently, knockdown of RNF26 inhibited the expression of *IFNB1* gene in various cells at the early phase and promoted it at the late phase of viral infection, respectively. Furthermore, knockdown of RNF26 inhibited viral replication, indicating that RNF26 antagonizes cellular antiviral response. Our findings thus suggest that RNF26 temporally regulates innate antiviral response by two distinct mechanisms.

## Author Summary

Virus infection induces the host cells to produce type I interferons, which are secreted proteins important for the host to clear viruses. Previously, we identified a cellular protein called MITA, which is essential for virus-triggered induction of interferons. In this study, we found an enzyme called RNF26 could covalently modify MITA with one type of polypeptide, called polyubiquitin. This modification caused increased stability of MITA after viral infection. RNF26 also caused disability of IRF3, another important component required for virus-triggered interferon induction. Thus, RNF26 could temporally regulate virus-triggered interferon induction by two distinct mechanisms. This discovery helps to understand how the antiviral response is delicately regulated.

## Introduction

Host pattern-recognition receptors (PRRs) detect nucleic acid from invading viruses or necrotic cells and trigger a series of signaling events that lead to the induction of type I interferons (IFNs), which plays a central role in autoimmune diseases as well as protective immune responses against viruses, respectively [1], [2]. Much progress has been made to characterize viral nucleic acid-triggered signaling pathways that result in transcriptional activation of type I IFN genes. A family of DExD/H box RNA helicases consisting of retinoic acid inducible gene I (RIG-I), melanoma differentiation-associated gene 5 (MDA5), and LGP2 are RNA sensors and recruit the adaptors VISA (also called MAVS, IPS-1 and Cardif) [3]–[6] and MITA (also known as STING, MPYS and ERIS) [7]–[10]to activate the transcription factors NF-κB and interferon regulatory factor (IRF) 3 or IRF7, leading to transcriptional induction of the genes encoding type I IFNs and other antiviral effectors [2]. A number of DNA sensors have been identified, including DAI, IFI16, DDX41 and MRE11 which may function in a ligand- and/or cell-type-specific manner [11]–[14]. In addition, cyclic GMP-AMP synthase (cGAS) has been recently characterized as a viral DNA sensor in almost all types of cells [15]. These sensors depend exclusively on the adaptor MITA to activate NF-κB and IRF3/7 and lead to subsequent induction of type I IFNs [16].

MITA is localized to the endoplasmic reticulum (ER), mitochondria-associated membrane and mitochondria [7], [8], [10], [17]. Upon viral infection, MITA translocates to intracellular membrane-containing compartments to form punctate aggregates and acts as a scaffold protein to facilitate the phosphorylation of IRF3 and STAT6 by the kinases TBK1 and IKKε [18]–[21]. Recent studies also suggest that MITA is a direct sensor that recognizes cyclic dinucleotides such as c-di-AMP, c-di-GMP and cGAMP generated from self and viral DNA infection [22]–[24].

It has been demonstrated that MITA undergoes various post-translational modifications and such modifications are key to the activity and stability of MITA [7], [21], [25]–[27]. MITA is phosphorylated at Ser358 by TBK1 which is critical for phosphorylation and activation of IRF3 [7], while UNC-51-like kinase (ULK1) phosphorylates MITA at Ser366 which impairs MITA-IRF3 interaction and subsequent activation of IRF3 [21]. In addition, RNF5 catalyzes K48-linked polyubiquitination of MITA and targets MITA for degradation [25], whereas TRIM56 and TRIM32 promote K63-linked polyubiquitination of MITA and positively regulates virus-triggered type I IFN induction [26], [27]. Whether and how additional proteins mediate other types of modifications of MITA is unknown.

In the present study, we identified an E3 ubiquitin ligase, RING finger protein 26 (RNF26) that targeted MITA for K11-linked polyubiquitination upon viral infection. MITA with K11-linked polyubiquitin chains remained as reservoir of MITA and was protected from RNF5-mediated K48-linked polyubiquitination and degradation. However, overexpression of RNF26 indirectly induced degradation of IRF3 through an autophagy pathway. As a result, knockdown of RNF26 promoted degradation of MITA after viral infection and prevented degradation of IRF3. Virus-triggered phosphorylation of IRF3 and induction of IFN-β were inhibited at the early time points and potentiated at late time points in the absence of RNF26, respectively. Our findings suggest that RNF26 temporally regulates virus-triggered induction of type I IFNs by two distinct mechanisms.

## Results

### RNF26 is an E3 ubiquitin ligase for MITA

**Figure 1:**
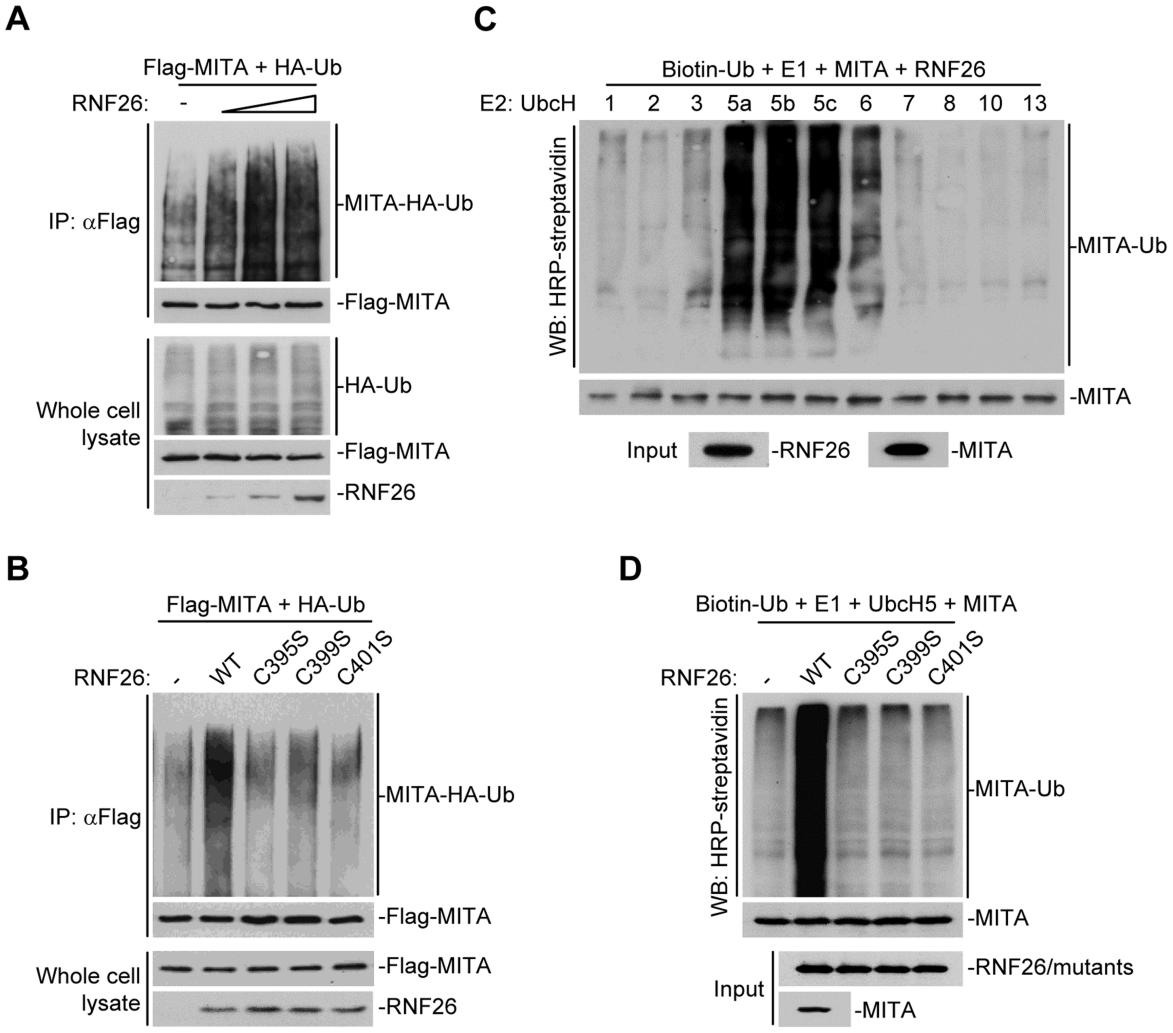
RNF26 is an E3 ubiquitin ligase for MITA. (A) Overexpression of RNF26 promoted polyubiquitination of MITA. The 293 cells (5×10^6^) were transfected with Flag-MITA (5 µg) and HA-Ub (1 µg) together with a control or RNF26 expression plasmid (0.5, 1, 2 µg). Twenty-four hours after transfection, cells were subjected to immunoprecipitation (IP) under denatured conditions with anti-Flag and the immunoprecipitates were analyzed by immunoblots with anti-HA (upper panel) or anti-Flag (lower panel). The whole cell lysates were analyzed by immunoblots with anti-HA (upper panel), anti-Flag (middle panel) and anti-RNF26 (bottom panel). Ub, ubiquitin. (B) RNF26-mediated polyubiquitination of MITA depends on the enzymatic activity of RNF26. The 293 cells (5×10^6^) were transfected with Flag-MITA (5 µg) and HA-Ub (1 µg) together with RNF26 or its mutants (1 µg each). IP under denatured conditions and ubiquitination assay was performed as described in (A). (C) UbcH5 mediated polyubiquitination of MITA by RNF26. The RNF26 and MITA proteins were obtained by *in vitro* transcription and translation, then incubated with biotin-Ub, E1 and the indicated E2s. Polyubiquitination of MITA was examined by immunoblot analysis with HRP-streptavidin (top panel). The inputs of RNF26 and MITA were analyzed by immunoblots with anti-MITA and anti-RNF26 (bottom panels). (D) RNF26 but not its enzymatic inactive mutants targeted MITA for polyubiquitination *in vitro*. MITA, RNF26 and its mutants were obtained by *in vitro* transcription and translation. Biotin-Ub, E1, UbcH5 and MITA were incubated with RNF26 or its mutants, followed by ubiquitination and immunoblot analysis as described in (C). All experiments were repeated for at least three times with similar results.

Because post-translational modifications of MITA are critical for mediating viral nucleic acid-triggered type I IFN induction, we assumed there are additional proteins that interact with and target MITA for various modifications and thereby regulate innate antiviral signaling. We attempted to unambiguously identify E3 ubiquitin ligases that regulate MITA ubiquitination and function [27]. This effort led to the identification of RNF26 which could promote polyubiquitination of MITA [27]. Analysis of the NCBI EST profile database indicates that RNF26 is ubiquitously expressed in most examined cells and tissues. In overexpression experiments, RNF26 dose-dependently promoted polyubiquitination of MITA (Figure 1A). In contrast, the enzymatic inactive mutants RNF26(C395S), RNF26(C399S) or RNF26(C401S) failed to mediate polyubiquitination of MITA (Figure 1B). Results from *in vitro* ubiquitination assays demonstrated that RNF26 catalyzed polyubiquitination of MITA *in vitro*, which depended on the enzymatic activity of RNF26 (Figure 1C–D). These data suggest that RNF26 is an E3 ubiquitin ligase targeting MITA for polyubiquitination.

### RNF26 interacts with MITA

**Figure 2:**
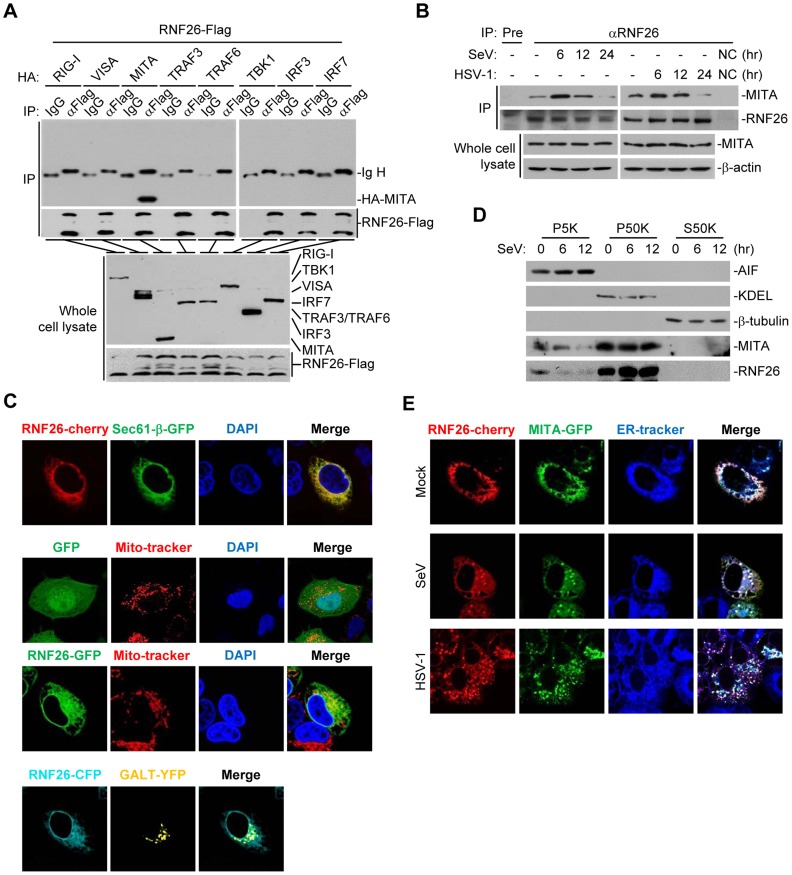
RNF26 interacts with MITA. (A) RNF26 interacted with MITA. The 293 cells (5×10^6^) were transfected with the indicated plasmids (5 µg each). Twenty-four hours later, cells were lysed and cell lysates were immunoprecipitated with anti-Flag or control IgG. The immunoprecipitates and whole cell lysates were analyzed by immunoblots with anti-HA or anti-Flag. (B) RNF26 was physiologically associated with MITA. THP-1 cells (2×10^7^) were infected with SeV or HSV-1 for the indicated time points or left uninfected. Cells were lysed and immunoprecipitation and immunoblot analysis was performed with antibodies against the indicated proteins. NC, negative control. (C) RNF26 is localized to the ER and mitochondria. HeLa cells (2×10^5^) were transfected with the indicated plasmids (0.5 µg each). Twenty-four hours after transfection, the cells were stained with Mito-Tracker Red for 15 minutes or left untreated. The cells were fixed with 4% paraformaldehyde and subjected for confocal microscopy analysis. (D) Subcellular distribution of RNF26. The 293 cells (5×10^7^) were infected with SeV for the indicated time points or left uninfected. Subcellular fractionation assays were performed and the cellular fractions were analyzed by immunoblots with antibodies against the indicated proteins. (E) RNF26 and MITA were colocalized to the ER. HeLa cells (2×10^5^) were transfected with the indicated plasmids (0.5 µg each). Eighteen hours after transfection, cells were infected with SeV or HSV-1 for 6 hours or left uninfected, followed by ER-Tracker Blue/White staining for 30 minutes. Cells were fixed with 4% paraformaldehyde and subjected for confocal microscopy analysis. All experiments were repeated for at least three times with similar results.

Since RNF26 caused polyubiquitination of MITA, we examined whether RNF26 interacted with MITA. Transient transfection and coimmunoprecipitation experiments indicated that RNF26 was associated with MITA in 293 cells (Figure 2A). In untransfected THP-1 cells, endogenous RNF26 constitutively interacted with MITA. This interaction was enhanced at 6 hours and decreased at 12–24 hours after SeV or HSV-1 infection (Figure 2B). It has been reported that MITA is localized to the ER, mitochondria-associated membrane and mitochondria [7], [8], [10], [17]. The subcellular localization of RNF26 was examined. Fluorescent confocal microscopy and cellular fractionation analysis suggested that RNF26 was mainly localized to the ER and a minor fraction of RNF26 was found to be colocalized with the mitochondria marker (Figure 2C and D). In contrast, RNF26 was not colocalized with the Golgi marker (Figure 2C). RNF26 was colocalized with MITA mostly at the ER and formed punctate dots with MITA after SeV or HSV-1 infection (Figure 2E). In this context, MITA was reported to translocate to microsomes to form punctate aggregates after viral infection or transfection of poly(dA:dT) or interferon stimulatory DNA (ISD) [17], [18], [20], [21]. These data suggest that RNF26 is physically associated with MITA.

Previously, we have demonstrated that the N-terminal transmembrane domains of MITA are critical for its subcellular localization and function [7]. Interestingly, sequence analysis indicated that RNF26 contained five transmembrane domains at the N-terminus and a RING domain at the C-terminus (Figure S1A). Domain mapping experiments indicated that the association of RNF26 and MITA depended on their respective transmembrane domains (Figure S1A and B). These data collectively suggest that RNF26 is physically associated with MITA and the interaction is dependent on their transmembrane domains.

### RNF26 promotes polyubiquitination of MITA at lysine 150 (K150)

**Figure 3:**
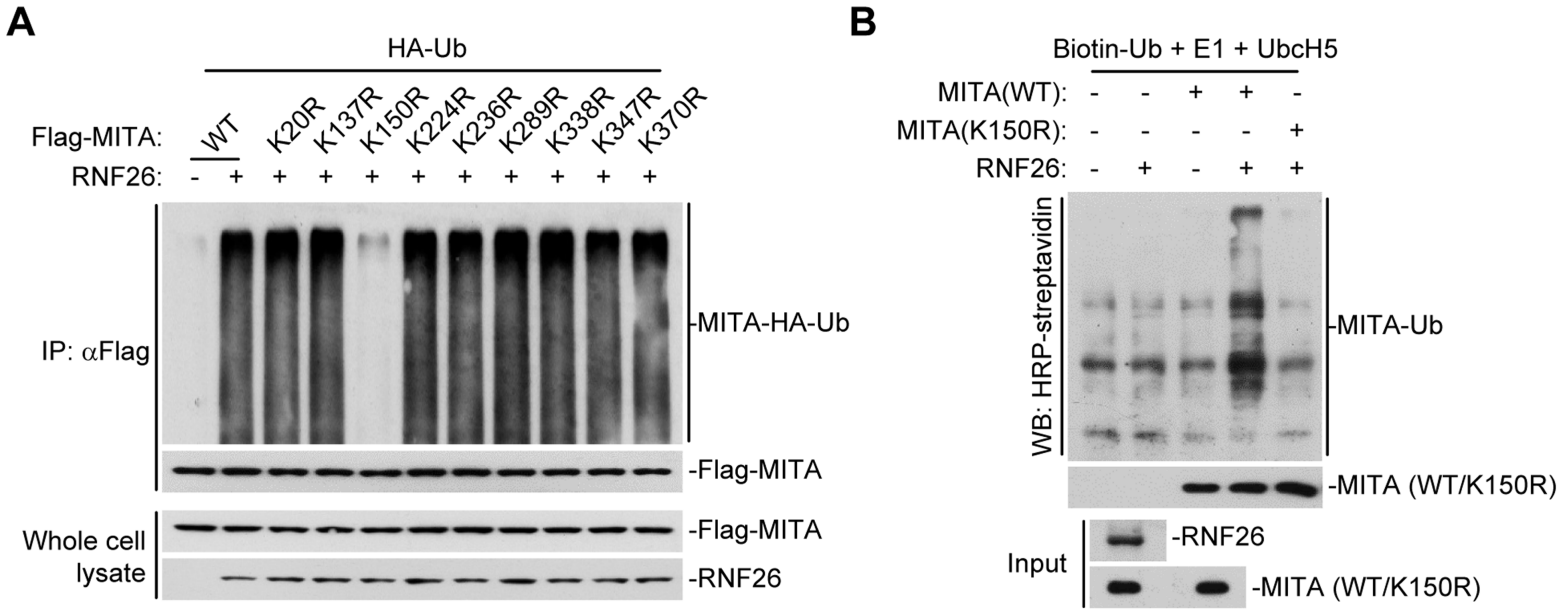
RNF26 promotes polyubiquitination of MITA at K150. (A) RNF26 mediated polyubiquitination of MITA at K150. The 293 cells (5×10^6^) were transfected with HA-Ub (1 µg) and RNF26 (1 µg) together with Flag-MITA or the indicated mutants (5 µg each). Twenty-four hours after transfection, cells were subjected IP under denatured conditions with anti-Flag and immunoprecipitates were analyzed by immunoblots with anti-HA (upper panel) or anti-Flag (lower panel). The whole cell lysates were analyzed by immunoblots with anti-Flag or anti-RNF26 as indicated. (B) RNF26 targeted MITA for polyubiquitination at K150 *in vitro*. RNF26, MITA and its mutants were obtained by *in vitro* transcription and translation. Biotin-Ub, E1, UbcH5 and RNF26 were incubated with MITA or its mutants. The ubiquitination of MITA was examined by immunoblot analysis with HRP-streptavidin (top panel). The inputs of RNF26 and MITA were analyzed by immunoblots with anti-MITA and anti-RNF26 (bottom panels). All experiments were repeated for at least three times with similar results.

To map the residue(s) of MITA that are targeted by RNF26, we examined RNF26-mediated polyubiquitination of MITA mutants in which all the lysine residues of MITA were individually substituted by arginine. As shown in Figure 3A, mutation of K150 to arginine impaired its polyubiquitination by RNF26. In addition, RNF26 could not induce polyubiquitination of MITA(K150R) in *in vitro* ubiquitination assays (Figure 3B). These data suggest that RNF26 targets K150 of MITA for polyubiquitination.

### RNF26 catalyzes K11-linked polyubiquitination of MITA

**Figure 4:**
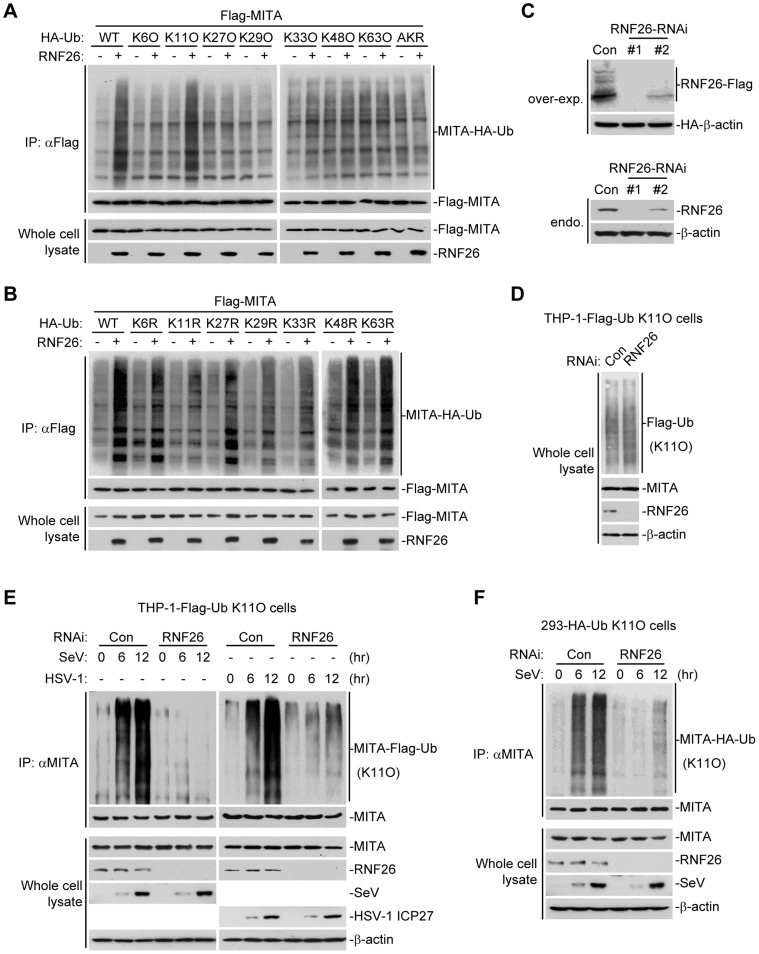
RNF26 catalyzes K11-linked polyubiquitination of MITA. (A and B) RNF26 targeted MITA for K11-linked polyubiquitination. The 293 cells (5×10^6^) were transfected with Flag-MITA (5 µg) and RNF26 (1 µg) together with HA-Ub or its mutants (1 µg each). Twenty-four hours after transfection, cells were subjected for IP under denatured conditions with limited amount of anti-Flag (0.5 µg) so that equal amount of Flag-MITA is pulled down. The immunoprecipitates were analyzed by immunoblots with anti-HA (upper panel) or anti-Flag (lower panel). The whole cell lysates were analyzed by immunoblots with anti-Flag or anti-RNF26 as indicated. Ub-AKR, all lysine residues of ubiquitin were mutated to arginine. (C) Effects of RNF26-RNAi plasmids on the expression of RNF26. In the upper panels, the 293 cells (1×10^6^) were transfected with the expression plasmids for RNF26-Flag (0.5 µg) and HA-β-actin (0.1 µg) together with the indicated RNAi plasmids (1 µg each). Twenty-four hours after transfection, whole cell lysates were analyzed by immunoblots with anti-Flag or anti-HA. In the lower panels, 293 cells were transduced with a control or RNF26-RNAi by retrovirus mediated gene transfer. Cells (1×10^6^) were lysed and whole cell lysates were analyzed by immunoblots with anti-RNF26 or anti-β-actin. (D) Immunoblot analysis of Flag-tagged ubiquitin expression in THP-1 cells stably transfected with Flag-Ub-K11O plasmid. Whole cell lysates of THP-1-Flag-Ub-K11O-RNF26-RNAi and control cells (1×10^6^) were analyzed by immunoblots with antibodies against the indicated proteins. RNF26-RNAi #1 was used here and in the following experiments if not noted. (E and F) Effects of RNF26 knockdown on virus-induced K11-linked polyubiquitination of endogenous MITA. In (E), THP-1-Flag-Ub-K11O-RNF26-RNAi or control cells (2×10^7^) were infected with SeV or HSV-1 for the indicated time points or left uninfected followed by IP under denatured conditions with anti-MITA. The immunoprecipitates were analyzed by immunoblots with anti-Flag (upper panels) or anti-MITA (lower panels). The whole cell lysates were analyzed by immunoblots with antibodies against the indicated cellular or viral proteins. In (F), 293-HA-Ub-K11O cells (2×10^7^) were transfected with a control or RNF26-RNAi plasmid (10 µg each). Twelve hours after transfection, puromycin (1 µg/mL) was added into the culture medium. The cells were selected for twenty-four hours and infected with SeV or left uninfected for the indicated time points followed by IP under denatured conditions and immunoblot analysis as in (E). All experiments were repeated for at least three times with similar results.

Having demonstrated that RNF26 is a MITA-interacting E3 ubiquitin ligase targeting MITA for polyubiquitination, the types of polyubiquitin chains conjugated to MITA by RNF26 were next examined. Ubiquitin mutants were constructed in which all but one lysine residues were simultaneously mutated to arginines (K-O) or all seven lysine residues were individually mutated to arginine (K-R). These mutants were examined for their abilities to be conjugated to MITA by RNF26. As shown in Figure 4A, the ubiquitin mutant retaining only lysine 11 (Ub-K11O) but not other lysine residues could be conjugated to MITA. Conversely, mutation of lysine 11 to arginine (Ub-K11R) markedly reduced its ability to be conjugated to MITA by RNF26 (Figure 4B). These data indicate that RNF26 induces K11-linked polyubiquitination of MITA.

Since an antibody against K11-linkage of polyubiquitin chains was not available to us, THP-1 cells stably transfected with Flag-Ub-K11O (THP-1-Flag-Ub-K11O) together with a control or RNF26-RNAi plasmids (Figure 4C and D) were established to examine viral infection-induced K11-linked polyubiquitination of MITA and the role of RNF26 in this ubiquitination. As shown in Figure 4E, MITA was modified with K11-linked polyubiquitin chains at 6–12 hours after SeV or HSV-1 infection, and such polyubiquitination was substantially impaired by knockdown of RNF26. Similar results were obtained in 293 cells stably transfected with HA-Ub-K11O plasmids (293-HA-Ub-K11O) together with a control or RNF26-RNAi plasmids (Figure 4F). In similar experiments, K11-linked polyubiquitination of VISA after viral infection was not affected by RNF26 knockdown (Figure S2). These data suggest that RNF26 mediates K11-linked polyubiquitination of MITA upon viral infection.

### RNF26 protects MITA from K48-linked polyubiquitination and degradation

**Figure 5:**
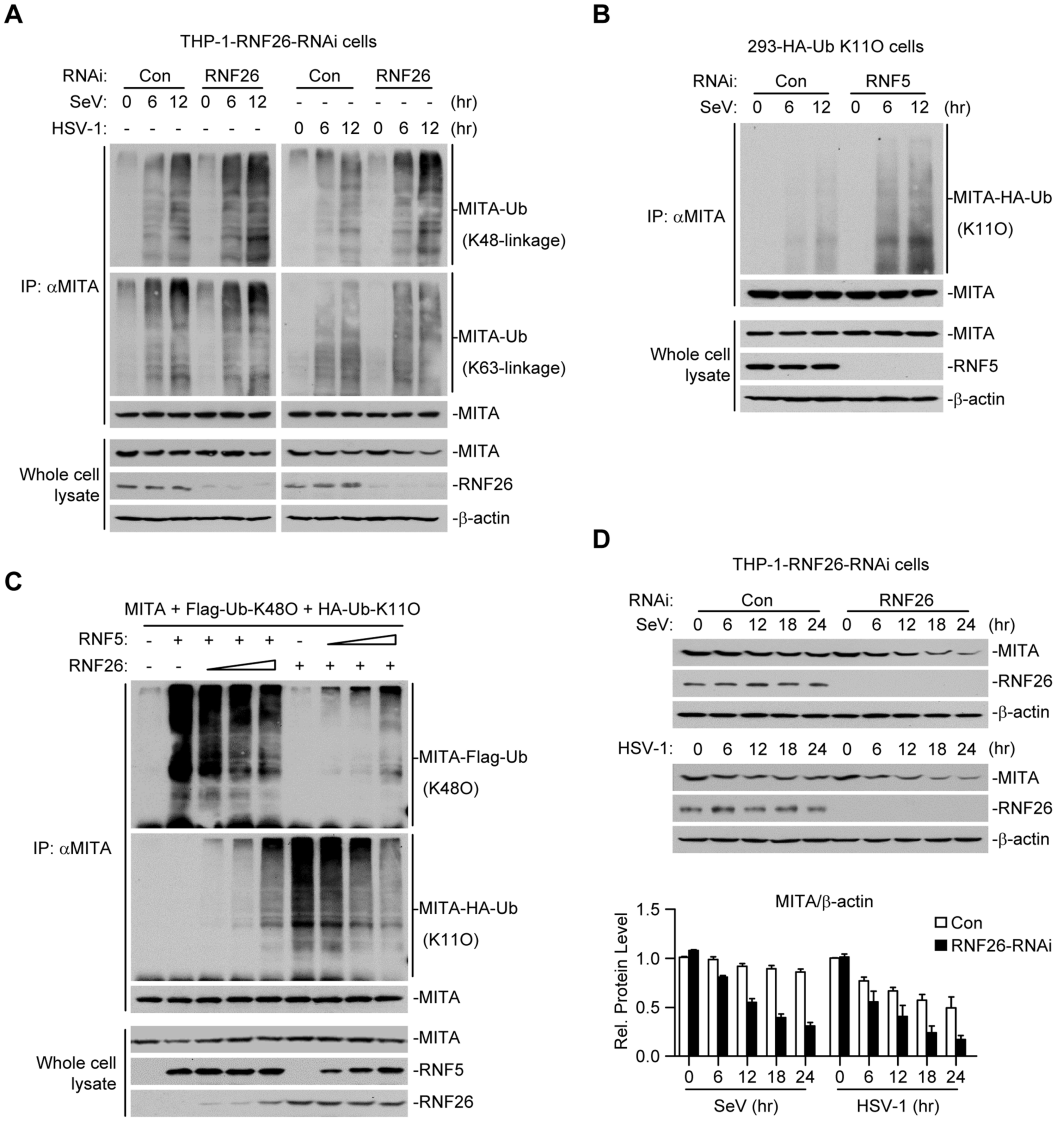
RNF26 protects MITA from K48-linked polyubiquitination and degradation. (A) Effects of RNF26 knockdown on virus-induced polyubiquitination of endogenous MITA. The THP-1-RNF26-RNAi or control cells (2×10^7^) were infected with SeV or HSV-1 for the indicated time points or left uninfected. Cell lysates were subjected to IP under denatured conditions with anti-MITA and the immunoprecipitates were analyzed by immunoblots with anti-Ub(K48) (upper panel), anti-Ub(K63) (middle panel) or anti-MITA (lower panel). The whole cell lysates were analyzed by immunoblots with antibodies against the indicated proteins. (B) Effects of RNF5 knockdown on virus-induced K11-linked polyubiquitination of endogenous MITA. The 293-HA-Ub-K11O cells (2×10^7^) were transfected with the indicated RNAi plasmid (10 µg each). Twelve hours after transfection, the cells were selected with puromycin (1 µg/mL) for twenty-four hours and infected with SeV for the indicated time points or left uninfected. Cell lysates were subjected to IP under denatured conditions with anti-MITA and the immunoprecipitates were analyzed by immunoblots with anti-HA (upper panel) or anti-MITA (lower panel). The whole cell lysates were analyzed by immunoblots with antibodies against the indicated antibodies. (C) RNF26 and RNF5 competed with each other on MITA polyubiquitination. The 293 cells (5×10^6^) were transfected with MITA (5 µg), Flag-Ub-K48O and HA-Ub-K11O (1 µg each) together with indicated amount of RNF26 and RNF5. Twenty-four hours after transfection, cell lysates were subjected to IP under denatured conditions with anti-MITA and the immunoprecipitates were analyzed by immunoblots with anti-Flag (upper panel), anti-HA (middle panel) or anti-MITA (lower panel). The whole cell lysates were analyzed by immunoblots with antibodies against the indicated proteins. (D) Effects of RNF26 knockdown on virus-triggered MITA degradation. The THP-1-RNF26-RNAi or control cells (1×10^6^) were infected with SeV or HSV-1 for the indicated time points or left uninfected. Cells were lysed and whole cell lysates were analyzed by immunoblots with antibodies against the indicated proteins (upper immunoblots). The relative protein levels of MITA in reference to β-actin were analyzed by Quantity One program and data shown are mean ± S.D. of three independent experiments (lower histographs).

Because multiple E3s have been reported to target polyubiquitin chains of distinct linkages (K48-linked or K63-linked) to K150 of MITA [25]–[27], we speculated that RNF26-mediated K11-linked polyubiquitination at K150 might compete with K48- or K63-linked polyubiquitination at the same lysine residue. Thus, virus-induced K48- or K63-linked polyubiquitination of MITA in THP-1-RNF26-RNAi and control cells was examined. As shown in Figure 5A, SeV or HSV-1 infection triggered K48- and K63-linked polyubiquitination of MITA. Knockdown of RNF26 potentiated K48- but not K63-linked polyubiquitination of MITA after viral infection (Figure 5A). RNF5 has been shown to induce K48-linked polyubiquitination of MITA at K150 [25]. Interestingly, we found that knockdown of RNF5 greatly enhanced SeV-induced K11-linked polyubiquitination of MITA in 293-HA-Ub-K11O cells (Figure 5B). In addition, RNF26-mediated K11-linked polyubiquitination of MITA was diminished by co-expression of RNF5, and RNF5-mediated K48-linked polyubiquitination and degradation of MITA was partially inhibited by co-expression of RNF26 (Figure 5C). Consistent with these observations, SeV- or HSV-1-induced degradation of MITA was accelerated by knockdown of RNF26 (Figure 5D). These data suggest that RNF26 catalyzes K11-linked polyubiquitination of MITA which protects MITA from RNF5-mediated K48-linked polyubiquitination.

### RNF26 modulates virus-triggered induction of type I IFNs

**Figure 6:**
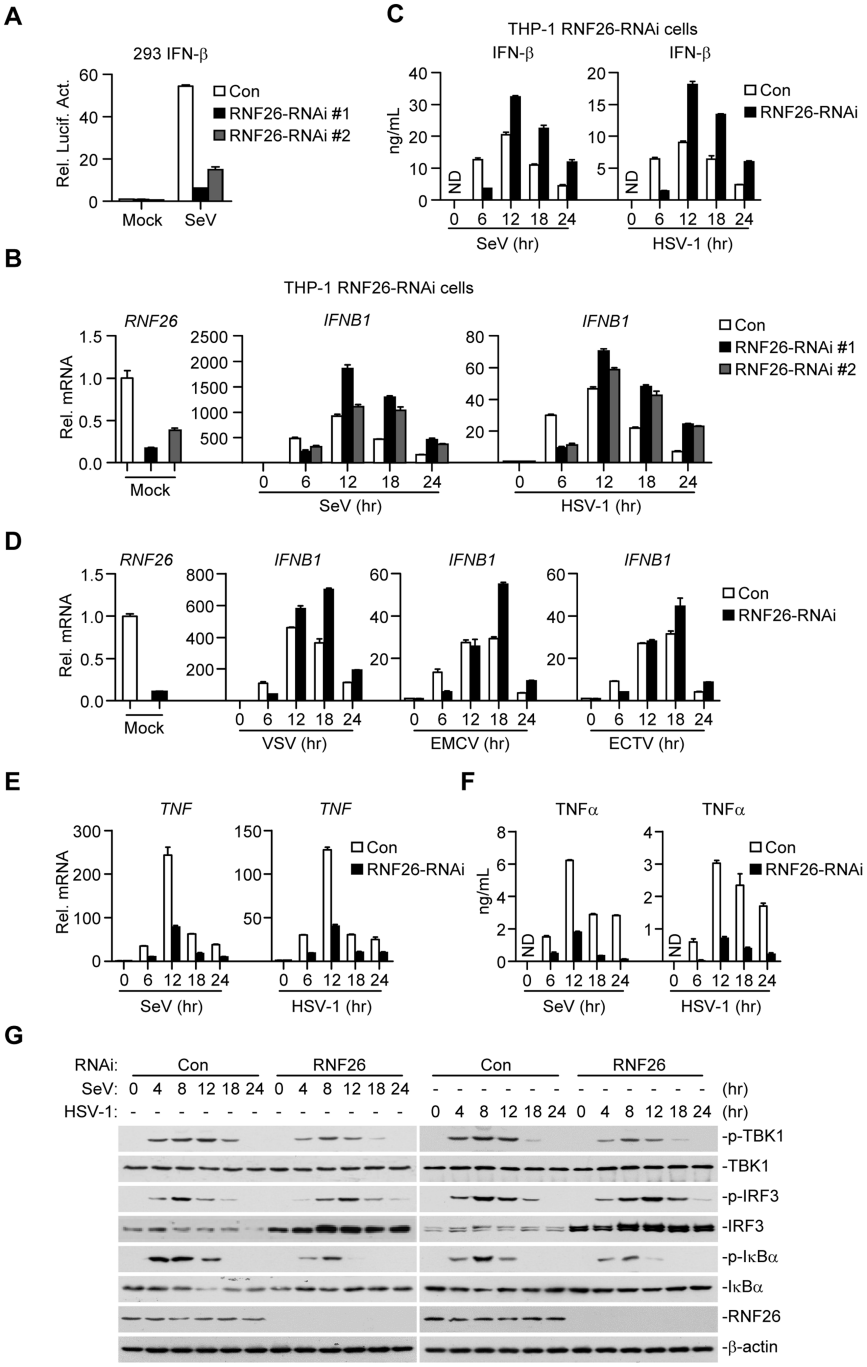
RNF26 modulates virus-triggered induction of type I IFNs. (A) Effects of RNF26 knockdown on SeV-triggered activation of the IFN-β promoter. The 293 cells (2×10^5^) were transfected with the IFN-β promoter reporter (0.1 µg) and the indicated RNAi plasmid (0.5 µg each). Thirty hours after transfection, cells were infected with SeV for 12 hours or left uninfected before reporter assays were performed. (B and C) Effects of RNF26 knockdown on virus-triggered induction of IFN-β in THP-1 cells. The THP-1-RNF26-RNAi or control cells (1×10^6^) were infected with SeV or HSV-1 for the indicated time points or left uninfected followed by quantitative real-time PCR (B) or ELISA analysis (C). ND, not detected. (D) Effects of RNF26 knockdown on virus-triggered induction of *IFNB1* gene in THP-1 cells. The THP-1-RNF26-RNAi or control cells (1×10^6^) were infected with VSV, EMCV or ECTV for the indicated time points or left uninfected before quantitative real-time PCR analysis was performed as in (B). (E and F) Effects of RNF26 knockdown on virus-triggered induction of TNFα in THP-1 cells. The THP-1-RNF26-RNAi or control cells (1×10^6^) were infected with SeV or HSV-1 for the indicated time points or left uninfected followed by quantitative real-time PCR (E) and ELISA analysis (F). (G) Effects of RNF26 knockdown on virus-triggered phosphorylation of TBK1, IRF3 and IκBα. The THP-1-RNF26-RNAi or control cells (1×10^6^) were infected with SeV or HSV-1 for the indicated time points or left uninfected, whole cell lysates were analyzed by immunoblots with anti-p-TBK1, anti-TBK1, anti-p-IRF3, anti-IRF3, anti-p-IκBα, anti-IκBα, anti-RNF26 or anti-β-actin as indicated. All experiments were repeated for at least three times with similar results. The bar graphs show mean ± S.D. (*n* = 3) of a representative experiment performed in triplicate.

Since RNF26-mediated K11-linked polyubiquitination of MITA protected its degradation after viral infection, whether RNF26 regulates virus-triggered induction of type I IFNs was determined. Luciferase reporter assays suggested that knockdown of RNF26 inhibited SeV-induced activation of NF-κB, ISRE and IFN-β promoter but had no marked effects on TNFα- or IL-1β-induced activation of NF-κB (Figure 6A and S3A). The expression of *IFNB1* gene was impaired at 6 hours after SeV infection by knockdown of RNF26 in 293 cells (Figure S3B). However, we unexpectedly observed that SeV-induced expression of *IFNB1* was potentiated in RNF26 knockdown cells compared to control cells at 12–24 hours after SeV infection (Figure S3B). In a mouse macrophage cell line Raw264.7 cells, SeV- or HSV-1-induced expression of *Ifnb1* gene was also inhibited and potentiated at the early and late time points by knockdown of murine Rnf26, respectively (Figure S3C and D). It should be noted that the degrees of inhibition or potentiation of expression of *IFNB1* or *Ifnb1* genes were correlated with the knockdown efficiencies of the RNF26-RNAi or Rnf26-RNAi plasmids, indicating that RNF26 is involved in regulating RNA and DNA virus-triggered induction of type I IFNs.

To further confirm this notion, RNAi-transduced stable THP-1 cell lines were established and the expression of *IFNB1* in these cells was examined after stimulation with SeV or HSV-1. As shown in Figure 6B and C, the mRNA and protein levels of IFN-β were impaired at 6 hours and potentiated at 12–24 hours in THP-1-RNF26-RNAi compared to control cells after viral infection, respectively. Similar results were obtained with various RNA (VSV and EMCV) or DNA (ECTV) viruses (Figure 6D) as well as virus-induced expression of *CCL5* (Figure S3E). Interestingly, SeV- or HSV-1-induced expression of the proinflammatory cytokines TNFα and IL-6 was inhibited by RNF26 knockdown at all examined time points after viral infection (Figure 6E, 6F, S4A and B). In similar experiments, RNF26 knockdown did not affect IFN-β-induced expression of *ISG15* or *ISG56* genes (Figure S4C). Consistent with the gene induction experiments, we found that although SeV- or HSV-1-induced phosphorylation of TBK1 and IκBα was inhibited in THP-1-RNF26-RNAi cells, phosphorylation of IRF3 was inhibited at the early time points and increased at the late time points in THP-1-RNF26-RNAi stable cells compared to that in control cells after viral infection (Figure 6G). Thus, we conclude that RNF26 temporally regulates virus-triggered induction of type I IFNs by two distinct mechanisms.

### RNF26 regulates IRF3 stability

**Figure 7:**
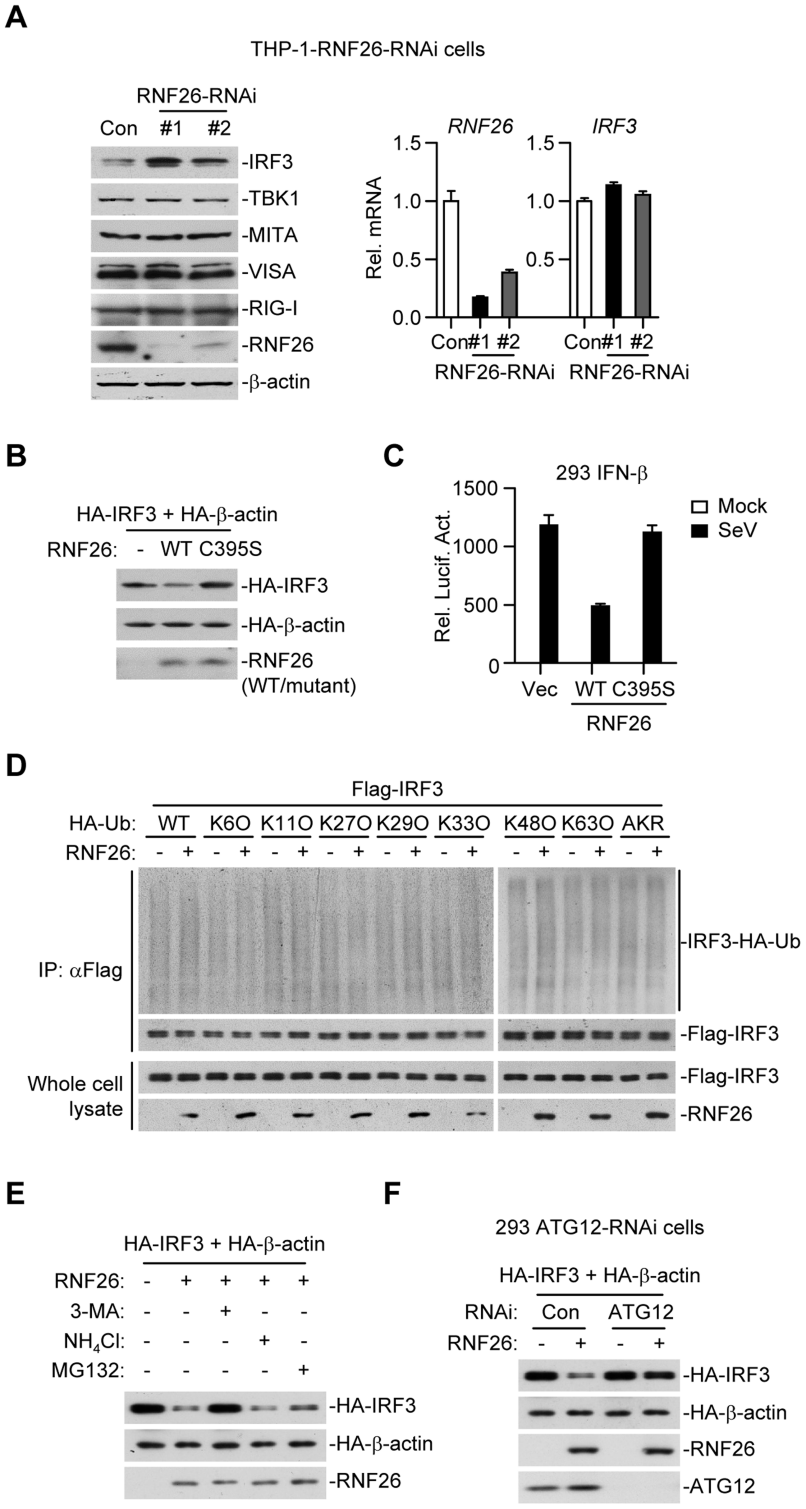
RNF26 regulates IRF3 stability. (A) Effects of RNF26 knockdown on IRF3 level. In the left panel, whole cell lysates of THP-1-RNF26-RNAi or control cells (1×10^6^) were analyzed by immunoblots with the indicated antibodies. In the right panel, cells (1×10^6^) were subjected to quantitative real-time PCR analysis. (B) Effects of RNF26 on IRF3 stability. The 293 cells (1×10^6^) were transfected with HA-IRF3 (0.5 µg) and HA-β-actin (0.1 µg) together with RNF26 or its mutant (0.3 µg each). Twenty-four hours later, whole cell lysates were analyzed by immunoblots with anti-HA or anti-RNF26. (C) Effects of RNF26 and its mutant on SeV-triggered activation of the IFN-β promoter. The 293 cells (2×10^5^) were transfected with the IFN-β promoter reporter (0.1 µg) and RNF26 or its mutant (0.1 µg each). Twenty hours after transfection, cells were infected with SeV for 12 hours or left uninfected before reporter assays were performed. (D) Effects of RNF26 on IRF3 ubiquitination. The 293 cells (5×10^6^) were transfected with Flag-IRF3 (5 µg) and RNF26 (1 µg) together with HA-Ub or its mutants (1 µg each). Eighteen hours after transfection, 3-MA (500 ng/mL) was added into culture medium for 4 hours to protect IRF3 from autophagosomal degradation during the experiment. The cell lysates were subjected to IP under denatured conditions with anti-Flag and the immunoprecipitates were analyzed by immunoblots with anti-HA (upper panels) or anti-Flag (lower panels). The whole cell lysates were analyzed by immunoblots with anti-Flag or anti-RNF26 as indicated. (E) Effects of inhibitors on RNF26-mediated destabilization of IRF3. The 293 cells (1×10^6^) were transfected with the indicated plasmids as described in (B). Eighteen hours after transfection, 3-MA (500 ng/mL), NH_4_Cl (25 mM) or MG132 (100 µM) was added into culture medium for four hours. Whole cell lysates were analyzed by immunoblots with anti-HA or anti-RNF26. (F) Effects of ATG12 knockdown on RNF26-mediated destabilization of IRF3. The 293-ATG12-RNAi or control cells (1×10^6^) were transfected with the indicated plasmids. Twenty-four hours later, whole cell lysates were analyzed by immunoblots with anti-HA, anti-RNF26 or anti-ATG12 as indicated. All experiments were repeated for at least three times with similar results. The bar graphs show mean ± S.D. (*n* = 3) of a representative experiment performed in triplicate.

When examining virus-triggered activation of IRF3 in RNF26 knockdown and control cells, we observed that the level of IRF3 protein was raised in THP-1-RNF26-RNAi compared to control cells (Figure 6G and 7A), and the mRNA levels of IRF3 were comparable in these cells (Figure 7A), indicating that RNF26 regulates IRF3 at the protein level. In support of this notion, we found that overexpression of RNF26 but not RNF26(C395S) promoted degradation of IRF3 and inhibited SeV-induced activation of the IFN-β promoter (Figure 7B and C). However, we failed to observe an interaction between IRF3 and RNF26 (Figure 2A) or polyubiquitination of IRF3 by RNF26 (Figure 7D). Interestingly, RNF26-mediated degradation of IRF3 was blocked by the autophagy inhibitor 3-methyladenine (3-MA) but not the lysosome inhibitor ammonium chloride (NH_4_Cl) or the proteasome inhibitor MG132 (Figure 7E). To further determine whether autophagic degradation system is responsible for RNF26-mediated degradation of IRF3, we determined the effect of knockdown of ATG12, an important component of the autophagic degradation system [28], on RNF26-mediated IRF3 degradation. The results indicated that RNF26-mediated IRF3 degradation was markedly inhibited in ATG12 knockdown cells (Figure 7F). Thus, RNF26 might temporally regulate virus-triggered type I IFN induction through regulating K11-linked polyubiquitination of MITA at the early phase and autophagy-dependent degradation of IRF3 at late phase, respectively.

### RNF26 functions as a negative regulator in cellular antiviral response

Since RNF26 regulates virus-induced expression of IFN-β and other downstream genes, we examined its roles in cellular antiviral response. We found that knockdown of RNF26 inhibited VSV and HSV-1 replication in plaque assays (Figure S5), suggesting that RNF26 functions as a negative regulator in cellular antiviral response.

## Discussion

MITA plays critical roles in virus-triggered type I IFN induction and innate antiviral immune response [7], [8], [10], [15], [17], [22]–[24]. Various studies have shown that post-translational modifications of MITA are essential for its function [7], [21], [25]–[27]. In this study, we demonstrated that RNF26 but not the enzymatic inactive mutants induced polyubiquitination of MITA. RNF26 mediated K11-linked polyubiquitination of MITA and modulated expression of type I IFN triggered by viral infection. RNF26 was localized mainly at the ER and constitutively interacted with MITA through their respective transmembrane domains. Viral infection potentiated this association and induced RNF26 to form punctate dots with MITA. Our studies suggest that RNF26 is a MITA-interacting E3 ubiquitin ligase which targets MITA for K11-linked polyubiquitination.

Polyubiquitination of MITA catalyzed by RNF26 was mapped to K150, which is also targeted by RNF5 for K48-linked polyubiquitination and by TRIM56 or TRIM32 for K63-linked polyubiquitination [25]–[27]. These observations prompted us to hypothesize that polyubiquitin chains of distinct linkages might compete with each other at the same residue of MITA. Interestingly, virus-triggered K48- but not K63-linked polyubiquitination of MITA was enhanced by knockdown of RNF26. Previously, it has been demonstrated that TRIM32 targets not only K150 but also K20, 224 and 236, whereas RNF5 targets only K150 of MITA [27]. This is consistent with our observations that RNF26 impaired K48- but not K63-linked polyubiquitination of MITA. RNF26-mediated K11-linked polyubiquitination of MITA protected it from RNF5-mediated K48-linked polyubiquitination and degradation. Consistently, degradation of MITA was accelerated in RNF26 knockdown cells compared to control cells after viral infection. These results suggest that RNF26-meidated K11-linked polyubiquitination competes with RNF5-mediated K48-linked polyubiquitination of MITA at K150. These results are consistent with our observations that knockdown of RNF26 inhibited induction of type I IFNs at early phase of viral infection. The functions of K11-linked polyubiquitination are not well understood so far [29]–[33]. To our knowledge, our study represents the first report on the function of K11-linked polyubiquitination in virus-triggered signaling and innate antiviral response.

**Figure 8:**
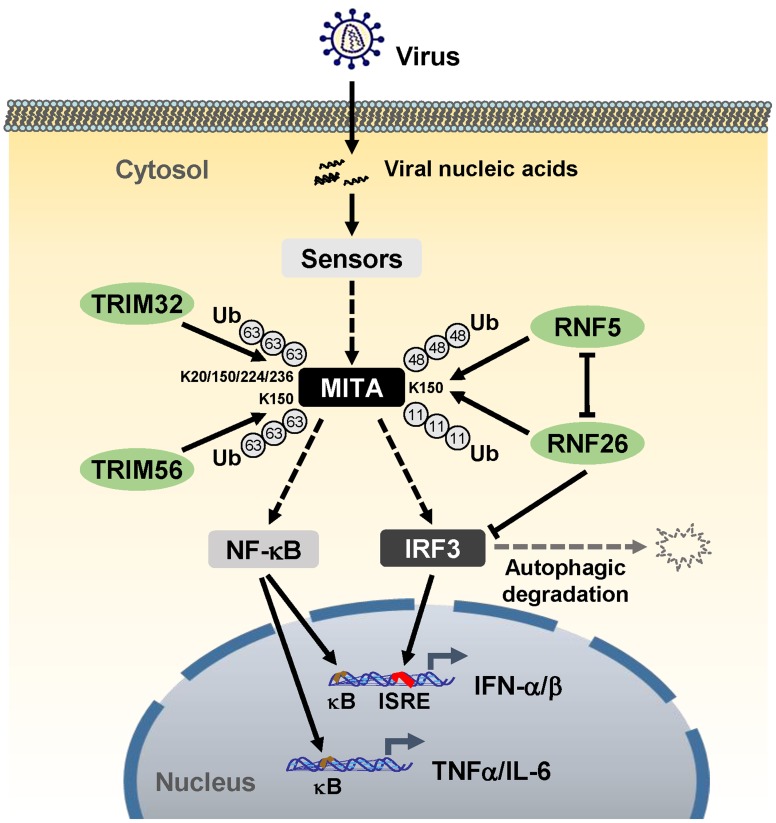
A model on the roles of RNF26 in virus-triggered induction of downstream genes. See text for details.

Unexpected, although knockdown of RNF26 inhibited virus-triggered induction of type I IFNs at the early phase of viral infection, it had opposite effect at the late phase of viral infection. This led us to hypothesize that RNF26 temporally regulates virus-triggered type I IFN induction by distinct mechanisms. In this context, we observed that overexpression of RNF26 promoted degradation of IRF3, whereas knockdown of RNF26 increased the level of IRF3. Interestingly, RNF26-mediated degradation of IRF3 was blocked by the autophagy inhibitor 3-MA but not the lysosome inhibitor NH_4_Cl or the proteasome inhibitor MG132. In addition, RNF26-induced degradation of IRF3 was markedly inhibited by knockdown of ATG12, an essential component in the autophagic degradation pathway. These results indicate that RNF26 indirectly regulates stability of IRF3 protein in an autophagy-dependent manner. The exact mechanism on how RNF26 mediates autophagy-dependent degradation of IRF3 is currently unknown. Based on our findings, we come to a working model on how RNF26 temporally regulates virus-triggered type I IFN induction (Figure 8). At the early phase of infection, RNF26 mediates K11-linked polyubiquitination of MITA, which protects it from K48-linked polyubiquitination and degradation to facilitate the fast induction of type I IFN genes. In addition to its early phase function through K11-linked polyubiquitination of MITA, RNF26 constitutively down-regulates IRF3 level by autophagic degradation. This may contribute to the termination of type I IFN induction at the late phase of viral infection. Interestingly, since IRF3 activation is not required for virus-triggered induction of the proinflammatory cytokines such as TNFα and IL-6, RNF26 positively regulates virus-triggered induction of the proinflammatory cytokines in a constitutive but not temporal manner. Therefore, our findings not only reveal the mechanisms on how RNF26 temporally modulate virus-triggered type I IFN induction, but also provide an explanation on how virus-triggered induction of type I IFNs and proinflammatory cytokines can be distinctly regulated.

## Materials and Methods

### Reagents and antibodies

Recombinant IFN-β, TNFα and IL-1β (R&D Systems); mouse monoclonal antibodies against FLAG (Sigma), HA (Covance), β-actin (Sigma), AIF, KDEL (Santa Cruz Biotechnology), β-tubulin (Invitrogen), HSV-1 ICP27, ATG12 (Abcam), TBK1, p-TBK1 and p-IκBα (CST); rabbit polyclonal antibodies against ubiquitin, IRF3, p-IRF3 (Santa Cruz Biotechnology), polyubiquitin K48-linkage and K63-linkage (Millipore) were purchased from the indicated manufacturers. SeV, HSV-1, VSV, EMCV, ECTV, anti-SeV, anti-RIG-I, anti-VISA, anti-MITA anti-RNF5 and anti-IκBα sera were previously described [7], [25], [27], [34]. Rabbit anti-RNF26 was raised against recombinant human RNF26 (241–433).

### Constructs

IFN-β, ISRE and NF-κB luciferase reporter plasmids, mammalian expression plasmids for HA-, Flag-, or GFP-tagged MITA and its mutants, ubiquitin, RIG-I, VISA, TRAF3, TRAF6, TBK1, IRF3, IRF7, Sec61-β, GALT, RNF5 and β-actin were previously described [7], [25], [27], [34], [35]. Mammalian expression plasmids for human Flag-, GFP-, Cherry- or CFP-tagged RNF26 and its mutants were constructed by standard molecular biology techniques.

### Transfection and reporter assays

The cells were seeded and transfected the following day by standard calcium phosphate precipitation method or by FuGENE (Roche). Empty control plasmids were added to ensure that each transfection receives the same amount of total DNA. To normalize for transfection efficiency, pRL-TK *Renilla* luciferase reporter plasmids were added to each transfection. Luciferase assays were performed using a dual-specific luciferase assay kit (Promega). Firefly luciferase activities were normalized on the basis of *Renilla* luciferase activities.

### Immunoprecipitation under denatured conditions and ubiquitination assay

The cells were lysed in lysis buffer containing 1% SDS and denatured by heating for 5 minutes. The supernatants were diluted with regular lysis buffer until the concentration of SDS was decreased to 0.1%. The diluted supernatants were subjected for immunoprecipitation as described [7], [25], [27], and the immunoprecipitates and whole cell lysates were analyzed by immunoblots with the indicated antibodies.

### *In vitro* ubiquitination assay

The tested proteins were expressed with a TNT Quick-coupled Transcription/Translation Systems kit (Promega) following instructions of the manufacturer. Ubiquitination was analyzed with an ubiquitination kit (Enzo Life Science) following protocols recommended by the manufacturer.

### Fluorescent confocal microscopy

The transfected cells were incubated with the ER-Tracker Blue/White or Mito-Tracker Red (Invitrogen) following protocols recommended by the manufacturer. The cells were then fixed with 4% paraformaldehyde for 10 minutes and observed with an Olympus confocal microscope under a ×60 oil objective.

### Subcellular fractionation

The cells were washed with PBS and lysed by douncing 30 times in 2 mL of homogenization buffer (10 mM Tris-HCl pH 7.4, 2 mM MgCl_2_, 10 mM KCl and 250 mM sucrose) on wet ice. The homogenate was centrifuged at 500×g for 10 minutes twice. The supernatant (S5) was centrifuged at 5,000×g for 30 minutes to precipitate crude mitochondria (P5K). The supernatant (S5K) was further centrifuged at 50,000×g for 60 minutes to generate S50K and P50K.

### RNAi experiments

Double-stranded oligonucleotides corresponding to the target sequences were cloned into the pSuper.Retro RNAi plasmids (oligoengine Inc.). The following sequences were targeted for human RNF26 cDNA: #1: 5′-GAGCAAGAGGAGCGGAAGA-3′; #2: 5′-GAGAGGATGTCATGCGGCT-3′. The following sequences were targeted for murine Rnf26 cDNA: #1: 5′-GAGCGGAAGAAGTGTGTTA-3′; #2: 5′-GATCAACAGTCTAGTCAAC-3′.

### Quantitative real-time PCR

Total RNA was isolated from cells using TRIzol reagent (Takara) and subjected to real-time PCR analysis to measure expression of mRNA. Gene-specific primer sequences were as described [27] or as follow: Human RNF26 (Forward: 5′-CAGGACCATCAGAGTGACACCT-3′; Reverse: 5′-GCAACACTGTCTTGCTCTGGTC-3′). Murine Rnf26 (Forward: 5′-TGGCTGCTTTCCTCGCTCACAT-3′; Reverse: 5′-GCAACACCAATCCAGTGAGATGG-3′). Human IRF3 (Forward: 5′-TCTGCCCTCAACCGCAAAGAAG-3′; Reverse: 5′-TACTGCCTCCACCATTGGTGTC-3′). Murine Irf3 (Forward: 5′-CGGAAAGAAGTGTTGCGGTTAGC-3′; Reverse: 5′-CAGGCTGCTTTTGCCATTGGTG-3′).

### ELISA

The supernatants of cell culture medium were analyzed with a human IFN-β (PBL), human TNFα or human IL-6 (Boster) ELISA kit following protocols recommended by the manufacturers.

### Flag-tagged ubiquitin stable THP-1 cells

The 293 cells were transfected with two packaging plasmids (pGAG-Pol and pVSV-G) together with pMSCV-GFP-Flag-Ub-K11O retroviral plasmids by calcium phosphate precipitation. Twenty-four hours after transfection, cells were incubated with new medium without antibiotics for another twenty-four hours. The recombinant virus-containing medium was filtered with 0.22 µm filter (Millex) and then added into cultured THP-1 cells in the presence of polybrene (4 µg/mL). The infected cells were cultured for at least seven days and sorted by a flow cell sorter before additional experiments were performed.

### HA-tagged ubiquitin stable 293 cells

The 293 cells were transfected with pRK7-Neo-HA-Ub-K11O plasmid. Twenty-four hours after transfection, cells were selected with G418 (0.8 µg/mL). Single cell colonies were picked and identified by immunoblot analysis.

### RNAi-transduced stable 293, THP-1 and Raw264.7 cells

The 293 cells were transfected with two packaging plasmids (pGAG-Pol and pVSV-G) together with a control, RNF26-RNAi or Rnf26-RNAi retroviral plasmids respectively by calcium phosphate precipitation. Twenty-four hours after transfection, cells were incubated with new medium without antibiotics for another twenty-four hours. The recombinant virus-containing medium was filtered with 0.22 µm filter (Millex) and then added into cultured 293, Raw264.7 or THP-1 cells in the presence of polybrene (4 µg/mL). The infected cells were selected with puromycin (1 µg/mL for 293 and Raw264.7 cells or 0.5 µg/mL for THP-1 cells) for at least seven days before additional experiments were performed.

### Accession numbers

UniProtKB/Swiss-Prot accession numbers (parentheses) are indicated for proteins mentioned in text: RNF26 (Q9BY78), MITA (Q86WV6), RNF5 (Q99942), VISA (Q7Z434).

## Supporting Information

Figure S1 **Domain mapping of the interaction between RNF26 and MITA.** (A) Interaction between full-length MITA and RNF26 truncations. Upper panels: a schematic presentation of full-length RNF26, its truncation mutants, and their abilities to interact with MITA. Lower panels: the 293 cells (5×10^6^) were transfected with HA-MITA (5 µg) and RNF26-Flag or its mutants (5 µg each). Twenty-four hours later, cells were lysed and cell lysates were immunoprecipitated with anti-HA or control IgG. The immunoprecipitates and whole cell lysates were analyzed by immunoblots with the indicated antibodies. FL, full-length. (B) Interaction between full-length RNF26 and MITA truncations. The experiments were performed similar to (A). All experiments were repeated for at least three times with similar results.
(PDF)

Figure S2 **RNF26 does not affect K11-linked polyubiquitination of VISA.** THP-1-Flag-Ub-K11O-RNF26-RNAi or control cells (2×10^7^) were infected with SeV or HSV-1 for the indicated time points or left uninfected. The cell lysates were subjected to IP under denatured conditions with anti-VISA and the immunoprecipitates were analyzed by immunoblots with anti-Flag (upper panels) or anti-VISA (lower panels). The whole cell lysates were analyzed by immunoblots with antibodies against the indicated proteins. All experiments were repeated for at least three times with similar results.
(PDF)

Figure S3 **RNF26 modulates virus-trigged induction of IFN-β and downstream genes.** (A) Effects of RNF26 knockdown on the activation of ISRE and NF- κB. The 293 cells (2×10^5^) were transfected with ISRE or NF-κB reporter (0.1 µg each) together with a control or RNF26-RNAi plasmid (0.5 µg each). Thirty hours after transfection, cells were treated with SeV, TNFα or IL-1β for 12 hours or left untreated before reporter assays were performed. (B) Effects of RNF26 knockdown on SeV-triggered induction of *IFNB1* gene in 293 cells. The 293-RNF26-RNAi or control cells (1×10^6^) were stimulated with SeV for the indicated time points or left uninfected followed by quantitative real-time PCR analysis. (C) Effects of Rnf26-RNAi plasmids on the expression of murine Rnf26. In the left panel, the 293 cells (1×10^6^) were transfected with expression plasmids for murine Rnf26-Flag (0.2 µg) and HA-β-actin (0.1 µg) together with the indicated RNAi plasmids (1 µg each). Twenty-four hours after transfection, whole cell lysates were analyzed by immunoblots with anti-Flag or anti-HA. In the right panel, whole cell lysates of Raw264.7-Rnf26-RNAi or control cells (1×10^6^) were analyzed by immunoblots with the anti-RNF26 or anti-β-actin. (D) Effects of Rnf26 knockdown on virus-triggered induction of *Ifnb1* gene in Raw264.7 cells. The Raw264.7-Rnf26-RNAi or control cells (1×10^6^) were infected with SeV or HSV-1 for the indicated time points or left uninfected before quantitative real-time PCR analysis was performed. (E) Effects of RNF26 knockdown on virus-triggered induction of *CCL5* genes. The THP-1-RNF26-RNAi or control cells (1×10^6^) were infected with SeV or HSV-1 for the indicated time points or left uninfected before quantitative real-time PCR analysis was performed. All experiments were repeated for at least three times with similar results. The bar graphs show mean ± S.D. (*n* = 3) of a representative experiment performed in triplicate.
(PDF)

Figure S4 **Effects of RNF26 knockdown on virus-trigged induction of IL-6 or IFN-β-triggered induction of ISGs.** (A and B) Effects of RNF26 knockdown on virus-triggered induction of IL-6 in THP-1 cells. The THP-1-RNF26-RNAi or control cells (1×10^6^) were infected with SeV or HSV-1 for the indicated time points or left uninfected followed by quantitative real-time PCR (A) or ELISA (B) analysis. (C) Effects of RNF26 knockdown on IFN-β-triggered induction of *ISG15* and *ISG56* genes in THP-1 cells. The THP-1-RNF26-RNAi or control cells (1×10^6^) were infected with SeV or HSV-1 for the indicated time points or left uninfected followed by quantitative real-time PCR. All experiments were repeated for at least three times with similar results. The bar graphs show mean ± S.D. (*n* = 3) of a representative experiment performed in triplicate.
(PDF)

Figure S5 **Roles of RNF26 in cellular antiviral responses.** (A and B) Effects of RNF26 knockdown on virus replication. THP-1-RNF26-RNAi or control cells were infected with VSV or HSV-1 (MOI = 0.1). The supernatants were harvested 36 hours after infection for standard plaque assays. All experiments were repeated for at least three times with similar results. The bar graphs show mean ± S.D. (*n* = 3) of a representative experiment performed in triplicate.
(PDF)
