# Supplementary figures and images for: RNF26 Temporally Regulates Virus-Triggered Type I Interferon Induction by Two Distinct Mechanisms
Source: PLoS Pathog. 2014 Sep 25;10(9):e1004358. doi: 10.1371/journal.ppat.1004358 (PMC4177927; doi:10.1371/journal.ppat.1004358)

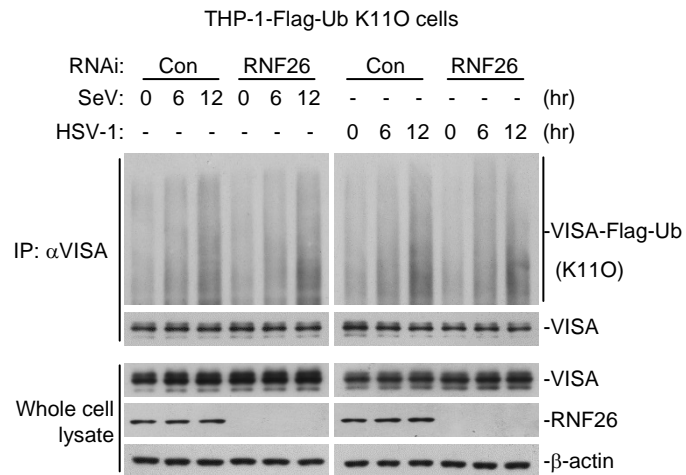

Supplement: Figure S2 — RNF26 does not affect K11-linked polyubiquitination of VISA. THP-1-Flag-Ub-K11O-RNF26-RNAi or control cells (2×10^7^) were infected with SeV or HSV-1 for the indicated time points or left uninfected. The cell lysates were subjected to IP under denatured conditions with anti-VISA and the immunoprecipitates were analyzed by immunoblots with anti-Flag (upper panels) or anti-VISA (lower panels). The whole cell lysates were analyzed by immunoblots with antibodies against the indicated proteins. All experiments were repeated for at least three times with similar results. (PDF) [file ppat.1004358.s002.pdf]

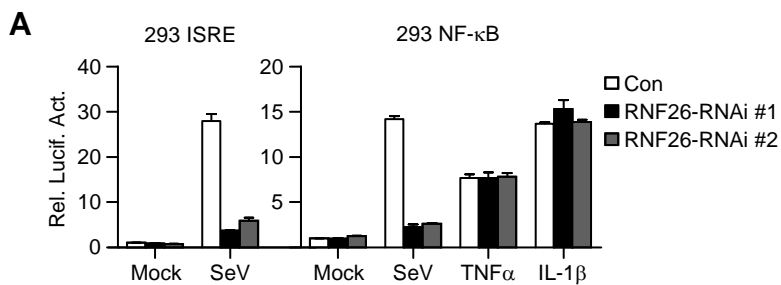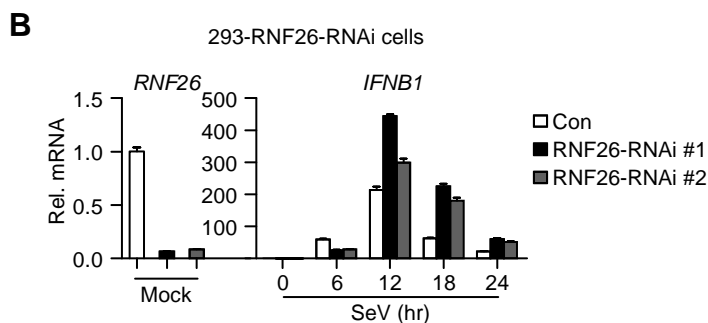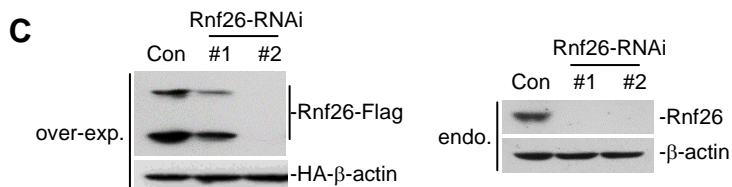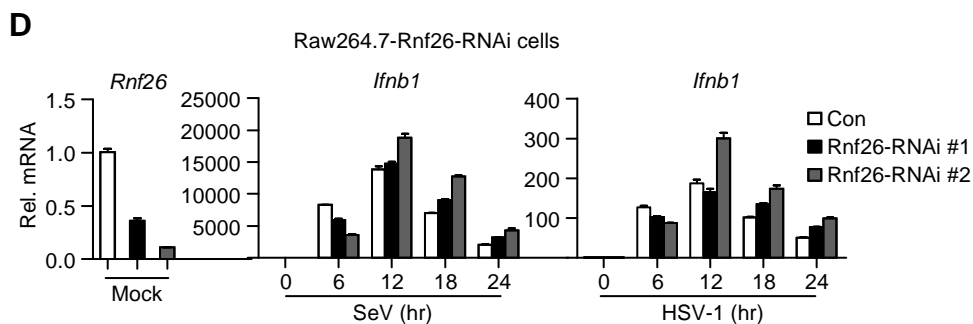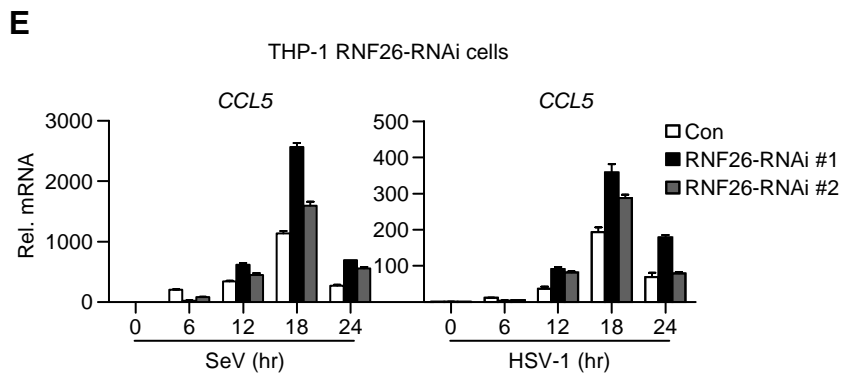

Supplement: Figure S3 — RNF26 modulates virus-trigged induction of IFN-β and downstream genes. (A) Effects of RNF26 knockdown on the activation of ISRE and NF- κB. The 293 cells (2×10^5^) were transfected with ISRE or NF-κB reporter (0.1 µg each) together with a control or RNF26-RNAi plasmid (0.5 µg each). Thirty hours after transfection, cells were treated with SeV, TNFα or IL-1β for 12 hours or left untreated before reporter assays were performed. (B) Effects of RNF26 knockdown on SeV-triggered induction of IFNB1 gene in 293 cells. The 293-RNF26-RNAi or control cells (1×10^6^) were stimulated with SeV for the indicated time points or left uninfected followed by quantitative real-time PCR analysis. (C) Effects of Rnf26-RNAi plasmids on the expression of murine Rnf26. In the left panel, the 293 cells (1×10^6^) were transfected with expression plasmids for murine Rnf26-Flag (0.2 µg) and HA-β-actin (0.1 µg) together with the indicated RNAi plasmids (1 µg each). Twenty-four hours after transfection, whole cell lysates were analyzed by immunoblots with anti-Flag or anti-HA. In the right panel, whole cell lysates of Raw264.7-Rnf26-RNAi or control cells (1×10^6^) were analyzed by immunoblots with the anti-RNF26 or anti-β-actin. (D) Effects of Rnf26 knockdown on virus-triggered induction of Ifnb1 gene in Raw264.7 cells. The Raw264.7-Rnf26-RNAi or control cells (1×10^6^) were infected with SeV or HSV-1 for the indicated time points or left uninfected before quantitative real-time PCR analysis was performed. (E) Effects of RNF26 knockdown on virus-triggered induction of CCL5 genes. The THP-1-RNF26-RNAi or control cells (1×10^6^) were infected with SeV or HSV-1 for the indicated time points or left uninfected before quantitative real-time PCR analysis was performed. All experiments were repeated for at least three times with similar results. The bar graphs show mean ± S.D. (n = 3) of a representative experiment performed in triplicate. (PDF) [file ppat.1004358.s003.pdf]

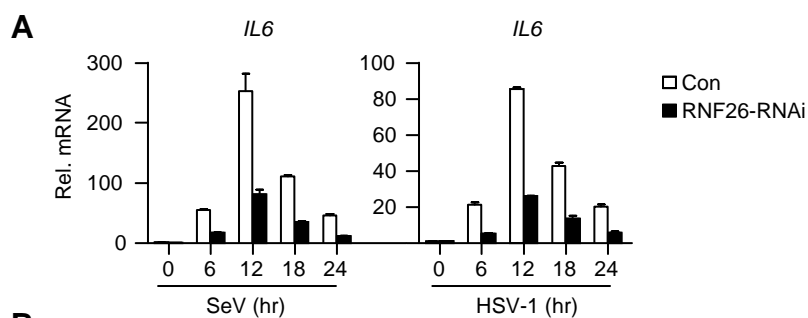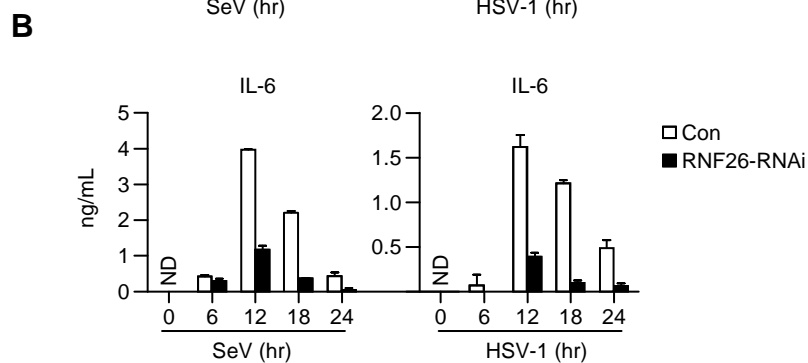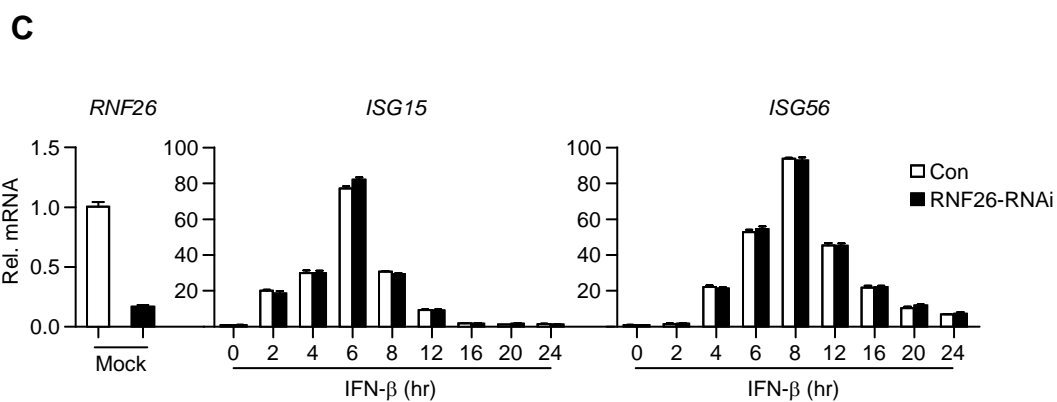

Supplement: Figure S4 — Effects of RNF26 knockdown on virus-trigged induction of IL-6 or IFN-β-triggered induction of ISGs. (A and B) Effects of RNF26 knockdown on virus-triggered induction of IL-6 in THP-1 cells. The THP-1-RNF26-RNAi or control cells (1×10^6^) were infected with SeV or HSV-1 for the indicated time points or left uninfected followed by quantitative real-time PCR (A) or ELISA (B) analysis. (C) Effects of RNF26 knockdown on IFN-β-triggered induction of ISG15 and ISG56 genes in THP-1 cells. The THP-1-RNF26-RNAi or control cells (1×10^6^) were infected with SeV or HSV-1 for the indicated time points or left uninfected followed by quantitative real-time PCR. All experiments were repeated for at least three times with similar results. The bar graphs show mean ± S.D. (n = 3) of a representative experiment performed in triplicate. (PDF) [file ppat.1004358.s004.pdf]

**A**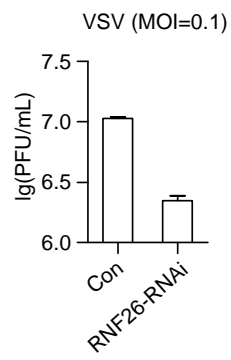**B**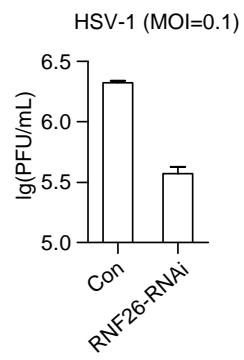

Supplement: Figure S5 — Roles of RNF26 in cellular antiviral responses. (A and B) Effects of RNF26 knockdown on virus replication. THP-1-RNF26-RNAi or control cells were infected with VSV or HSV-1 (MOI = 0.1). The supernatants were harvested 36 hours after infection for standard plaque assays. All experiments were repeated for at least three times with similar results. The bar graphs show mean ± S.D. (n = 3) of a representative experiment performed in triplicate. (PDF) [file ppat.1004358.s005.pdf]
