# Supplementary material for: RNF26 Temporally Regulates Virus-Triggered Type I Interferon Induction by Two Distinct Mechanisms
Source: PLoS Pathog. 2014 Sep 25;10(9):e1004358. doi: 10.1371/journal.ppat.1004358 (PMC4177927; doi:10.1371/journal.ppat.1004358)
Supplement: Figure S1 — Domain mapping of the interaction between RNF26 and MITA. (A) Interaction between full-length MITA and RNF26 truncations. Upper panels: a schematic presentation of full-length RNF26, its truncation mutants, and their abilities to interact with MITA. Lower panels: the 293 cells (5×10^6^) were transfected with HA-MITA (5 µg) and RNF26-Flag or its mutants (5 µg each). Twenty-four hours later, cells were lysed and cell lysates were immunoprecipitated with anti-HA or control IgG. The immunoprecipitates and whole cell lysates were analyzed by immunoblots with the indicated antibodies. FL, full-length. (B) Interaction between full-length RNF26 and MITA truncations. The experiments were performed similar to (A). All experiments were repeated for at least three times with similar results. (PDF) [file ppat.1004358.s001.pdf]

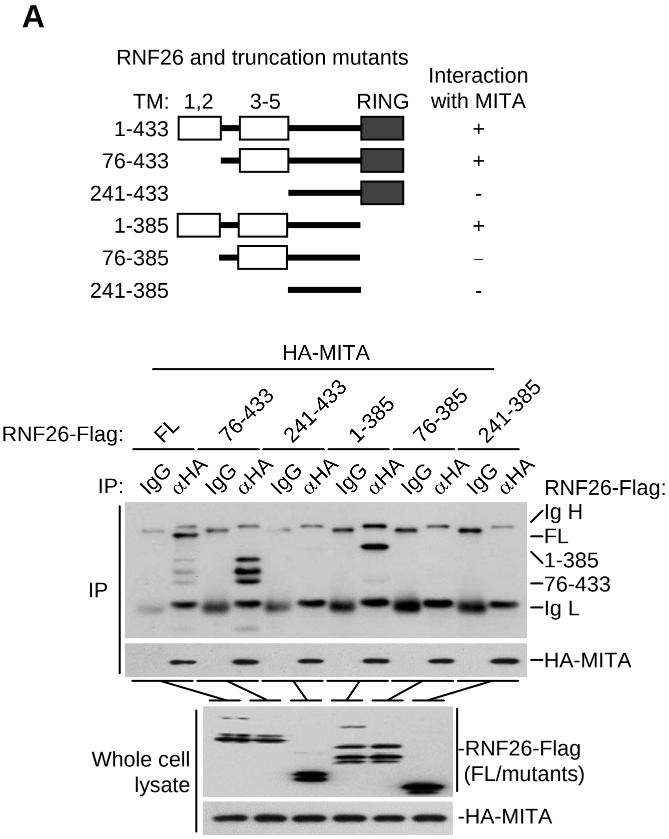
A
RNF26 and truncation mutants
Interaction with MITA
TM: 1,2 3-5 RING
1-433 +
76-433 +
241-433 -
1-385 +
76-385 –
241-385 -
HA-MITA
RNF26-Flag: FL 76-433 241-433 1-385 76-385 241-385
IP: IgG αHA IgG αHA IgG αHA IgG αHA IgG αHA IgG αHA
RNF26-Flag:
Ig H
FL
1-385
76-433
Ig L
IP
HA-MITA
Whole cell lysate
-RNF26-Flag (FL/mutants)
-HA-MITA

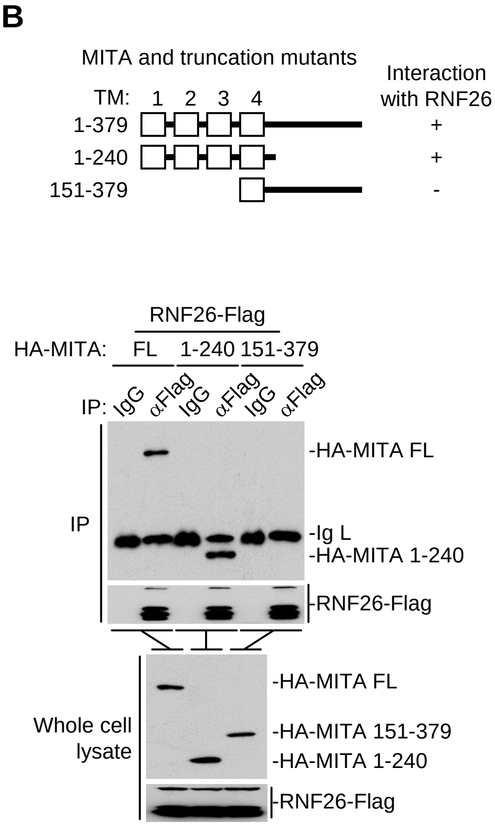
B
MITA and truncation mutants
Interaction with RNF26
TM: 1 2 3 4
1-379 +
1-240 +
151-379 -
RNF26-Flag
HA-MITA: FL 1-240 151-379
IP: IgG αFlag IgG αFlag IgG αFlag
-HA-MITA FL
IP
-Ig L
-HA-MITA 1-240
-RNF26-Flag
-HA-MITA FL
Whole cell lysate
-HA-MITA 151-379
-HA-MITA 1-240
-RNF26-Flag
